# Another Bureau Loss

**Published:** 1900-04

**Authors:** 


### ANOTHER BUREAU LOSS.

THE loss of Dr. Charles F. Dawson to the Bureau of Animal Industry is another one of those misfortunes which the Government is continually experiencing, owing to the small salaries accorded to expert skill and ability. Many of the scientific departments have long ago become the training schools for expert scientists eagerly sought for by commercial enterprises throughout the land. This should not be, as the years of special training in Government work, fitting men peculiarly for the most valuable work for our Government (our people), are thus lost to private enterprises by virtue of the meagre compensation given. While there are hundreds of overpaid workers in the Government employ, the compensation of many of the heads of scientific departments are very much underpaid, and those in authority should not be unmindful of the need in this direction, and why this should no longer be. The vast amount of scientific work done for our people and the valued assistance thus given should be enhanced and advanced, and this special work properly compensated for. More than 60 per cent. of the personnel of this department have been lost during the past ten years in this way.

